# Efficacy and safety of a colostrum- and *Aloe vera*-based oral care protocol to prevent and treat severe oral mucositis in patients undergoing hematopoietic stem cell transplantation: a single-arm phase II study

**DOI:** 10.1007/s00277-022-04934-4

**Published:** 2022-08-03

**Authors:** Monica Guberti, Stefano Botti, Cristiana Caffarri, Silvio Cavuto, Luisa Savoldi, Andrea Fusco, Francesco Merli, Michela Piredda, Maria Grazia De Marinis

**Affiliations:** 1 Research and EBP Unit, Health Professions Department, Azienda USL-IRCCS Di Reggio Emilia, Via Amendola 2, 42122 Reggio Emilia, Italy; 2 Department of Biomedicine and Prevention, University of Rome “Tor Vergata”, Via Montpellier, 1 - 00133 Rome, Italy; 3 Hematology Unit, Azienda USL-IRCCS Di Reggio Emilia, Via Amendola 2, 42122 Reggio Emilia, Italy; 4 Clinical Trials and Statistics Unit, SC Infrastructure, Research and Statistics, Azienda USL-IRCCS Di Reggio Emilia, Via Amendola 2, 42123 Reggio Emilia, Italy; 5 Research Unit Nursing Science, University Campus Bio-Medico of Rome, Via Alvaro del Portillo 21, 00128 Rome, Italy

**Keywords:** Hematopoietic stem cell transplantation, Colostrum, *Aloe vera*, Oral mucositis, Conditioning regimen, Oral toxicity

## Abstract

Oral mucositis is one of the worst effects of the conditioning regimens given to patients undergoing hematopoietic stem cell transplantation. It is characterized by dry mouth, erythema, mucosal soreness, ulcers, and pain, and it may impact patient outcomes. Bovine colostrum and *Aloe vera* contain a wide variety of biologically active compounds that promote mucosal healing. A non-randomized phase II study was designed to assess the safety and efficacy of a combined bovine colostrum and *Aloe vera* oral care protocol to prevent and to treat severe oral mucositis in transplant patients. Two commercially available products were given to patients in addition to the standard protocol: Remargin Colostrum OS® mouthwash and Remargin Colostrum Gastro-Gel® taken orally. Forty-six (78.0%) patients experienced oral mucositis, 40 (67.8%) developed mild–moderate forms, and 6 (10.2%) severe ones. Comparing the study group’s outcomes with those of a homogeneous historical control group, severe oral mucositis decreased significantly (10.2% vs. 28.4%; *P* < 0.01), as did its duration (0.5 ± 1.9 vs. 1.5 ± 3.0 days; *P* < 0.01). Febrile neutropenia episodes (69.5% vs. 95.1%; *P* < 0.01) and duration (4.0 ± 4.7 vs. 6.2 ± 4.5 days; *P* < 0.01) also decreased. These findings show that the experimental protocol seems effective in preventing severe forms of oral mucositis. However, a randomized controlled trial is necessary to confirm this.

## Background

Allogeneic and autologous hematopoietic stem cell transplantation (HSCT) are standards of treatment for several hematological malignancies [1–3]. Before stem cell infusion, the recipient is treated with a conditioning regimen that includes combinations of chemotherapy, radiotherapy, and/or immunotherapy [4, 5].

Chemotherapy- and/or radiotherapy-induced oral epithelial cell damage, known as oral mucositis (OM), is considered one of the worst toxic effects of conditioning regimens [6]. It is a predictable clinical condition favored also by some predisposing factors, including epigenetic, metabolomic, and microbiome-related ones [7–9], and it is experienced by the 70–100% of HSCT patients undergoing myeloablative conditioning regimens (MAC) [10–14]. Its signs and symptoms include dry mouth, taste and salivary change, erythema, mouth soreness, ulcers, and pain. Severe forms of OM (sOM) may impact patients’ quality of life (QoL) [15–20], as well as transplant-related morbidity and mortality, and healthcare costs [21–24].

The literature provides few evidence supporting strategies to prevent or treat OM [25, 26]. Thus, the approaches to dealing with OM in daily practice often rely on a wide variety of products supported by scarce or anecdotal evidence [27, 28]. An interest in using natural agents for OM has been increasing, since these products may be effective for symptom control and because their components can interfere with the pathobiological processes underlying OM development [29–31].

Bovine colostrum (BC) has a wide variety of biologically active components, including lactoferrin, lactoperoxidase, immunoglobulins, and growth factors, and its benefits on health as a dietary supplement have been widely studied [32–34]. The protective effects of BC on the intestinal mucosal barrier [35] and upper respiratory tract integrity [36–38] have been reported, as have its beneficial effects on boosting the immune system [39, 40]. In addition, topical applications of BC have been effective in both wound and mucosal healing thanks to its humectant, moisturizing, re-epithelizing, antioxidant, and immune-stimulant activities [41–44].

*Aloe vera* (AV)-based preparations contain various active compounds, including iron, folic acid, electrolytes, and vitamins, that have positive effects on general health [45–48]. Its formula shows emollient, moisturizing, anti-inflammatory, and immune-modulatory properties [49, 50], and has been studied for the prevention and treatment of several mucocutaneous conditions, without any adverse effects [51, 52].

Therefore, we hypothesized that combined formulas of BC and AV, in addition to the standard oral care practice, would effectively and safely prevent and treat sOM in patients undergoing HSCT.

## Materials and methods

### Study design and sample size

A single-arm, non-randomized, open label, single-center, phase II study was designed following the optimal two-stage design by Richard Simon [53]. Adult patients undergoing autologous or allogeneic HSCT were recruited; those who reported intolerance to the products’ components, who were not able to use the study self-reporting tools, or patients with OM already present at admission were excluded from the study. A study group (SG) sample size of 59 recipients was calculated assuming a reduction of 50% than local benchmarking data on sOM, and considering an *α* error of 0.05 and a sensitivity of 0.8. The study design provided a first step of 19 participants with a cutoff for study discontinuation of more than 5 patients with sOM. After recruitment, all patients received educational intervention (interview and educational material) on study medication management and the use of the tools included in the study. The study protocol was approved by the local ethics committee (n. 2016/0030535, December 28, 2016) and it was conducted in agreement with the Helsinki Declaration of 1975 and the Guidelines for Good Clinical Practice. All patients gave written informed consent before any study-related procedure took place.

### Oral care protocol

In the transplant unit where the study has been performed, a standard oral care protocol was used to prevent and treat OM. It includes oral hygiene, i.e., gentle cleansing with toothbrush and toothpaste, followed by bland saline rinses (normal saline or sodium bicarbonate), 3 times per day after each meal, with the frequency increasing after OM onset. In addition, mouthwashes with moisturizing and emollient solutions and lip balm were recommended to all patients.

Two products containing BC and AV were added to standard practice in this study: (1) Remargin Colostrum OS® (RCOS), 10-ml single-dose stick pack natural mouthwashes containing water, *Aloe barbadensis* leaf juice, colostrum, glycerine, seed extracts, vegetable oils, sucralose, potassium sorbate, and citric acid (Solimè srl, Cavriago, Reggio Emilia, Italy, Patent No. 1291340); (2) Remargin Colostrum Gastro-Gel® (RCGG), 4-g single-dose stick-pack dietary supplement containing water, *Aloe barbadensis* gel, colostrum, maltodextrin, sorbitol, seed extracts, vegetable oils, sodium alginate, potassium sorbate, citric acid, and pectin (Solimè srl, Cavriago, Reggio Emilia, Italy, Patent No. 920596386).

Patients performed RCOS mouthwashes (1 stick pack) for 40–60 s after each oral hygiene, and RCGG (1 stick pack) was administered orally 3 times per day from the start of conditioning until OM onset (prevention phase). After OM onset (treatment phase), the frequency of intervention was increased to at least 3 to 5 times per day.

### Endpoints and outcomes

The primary endpoint was the incidence of sOM (grade 3–4 WHO) during the study period. OM was assessed daily by the nursing staff starting from the first day of conditioning until day 21 post-transplant using the WHO scale. Secondary endpoints were the evaluation of overall OM incidence, its time of onset, and duration. OM-related pain scores were assessed daily using a 0–10 numerical rating scale (NRS). Neutropenia and febrile neutropenia (FN) duration (days) and FN events were recorded, as were some cost-related outcomes such as length of stay, antibiotic, antifungal and antiviral therapy, and days of opioid and parenteral nutrition. QoL was assessed weekly with EQ-5D-3L (not reported), and patient-reported data were collected using the Oral Mucositis Daily Questionnaire (OMDQ) (not reported). Adherence to the study protocol was monitored. Safety was assessed by collecting data on adverse events (AEs) and by monitoring blood cell count, hemoglobin, and serum levels of creatinine and bilirubin. Oral swab tests for infection were performed at admission and at day 8 post-transplant, while galactomannan serum levels were monitored weekly until day 28 post-transplant or discharge.

The study outcomes were compared with routinely collected local benchmarking data of a historical cohort of patients treated during the 22 months preceding the study start (Table 1). This cohort had undergone only the standard practice protocol to prevent and treat OM and was taken as the control group (CG). All the data were collected by the patients’ electronic clinical documentation and all the nurses assessing outcomes in both groups were routinely trained to use the assessment tools.

**Table 1:** Group’s description

			Control group *n* (%)	Study group *n* (%)	*P* value (test)
Patients	*n*		81	59	
	Age (mean ± SD)		54.2 ± 13.3	52.4 ± 12.0	
	Male		49 (60.5)	32 (54.2)	0.46 (C)
	Female		32 (39.5)	27 (45.8)	
Diagnosis	HL/NHL		34 (42.0)	19 (32.2)	0.54 (C)
	PD		30 (37.0)	29 (49.2)	
	AL		14 (17.3)	9 (15.3)	
	BMF		3 (3.7)	2 (3.4)	
Transplant type	Autologous		63 (77.8)	44 (74.6)	0.66 (C)
	Allogeneic		18 (22.2)	15 (25.4)	
		Sibling	11 (13.6)	8 (13.5)	0.65 (C)
		Haplo	7 (8.6)	7 (11.9)	
Stem cell source	Stem cells		77 (95.1)	57 (96.6)	0.65 (C)
	Bone marrow		4 (4.9)	2 (3.4)	
Cell product	Cryopreserved		64 (79.0)	45 (76.3)	0.69 (C)
	Fresh		17 (21.0)	14 (23.7)	
Conditioning regimens	MAC		77 (95.1)	56 (94.9)	0.97 (C)
	RIC		4 (4.9)	3 (5.1)	
	Autologous	Mel200	30 (37.1)	29 (49.2)	0.09 (C)
		FEAM	33 (40.7)	15 (25.4)	
	Allogeneic	TTF	4 (4.9)	7 (11.8)	0.29 (MW)
		Bu-Cy	5 (6.2)	4 (6.8)	
		Flu-Cy-Thi	2 (2.5)	2 (3.4)	
		TBF	3 (3.7)	1 (1.7)	
		Cy-Flu-Mel	0 (0.0)	1 (1.7)	
		Bu-Flu	4 (4.9)	0 (0.0)	
Immunosuppression	CsA/MTX		11 (13.6)	8 (13.5)	0.65 (C)
	SRL/MPA		7 (8.6)	7 (11.9)	
Growth factors	GCSF	Yes	63 (77.8)	45 (76.3)	0.83 (C)
		No	18 (22.2)	14 (23.7)	
	KGF (palifermin)	Yes	0 (0.0)	0 (0.0)	1.0 (C)
		No	81 (100.0)	59 (100.)	
Risk factors for OM	Alcohol abuse	Yes	1 (1.2)	0.0 (0.0)	1.0 (F)
		No	80 (98.8)	59 (100)	
	Tobacco	Yes	13 (16.0)	6 (10.2)	0.32 (C)
		No	68 (84.0)	53 (89.8)	
	Previous OM	Yes	18 (22.2)	10 (16.9)	0.44 (C)
		No	63 (77.8)	49 (83.1)	

*n* number; *SD* standard deviation; *C* Chi square test; *HL/NHL* Hodgkin lymphoma/non-Hodgkin lymphoma; *PD* plasma cell disorders; *AL* acute leukemia; *BMF* bone marrow failure; *MAC* myeloablative conditioning; *RIC* reduced intensity conditioning; *Mel 200* melphalan 200 mg/m^2^; *FEAM* fotemustine-etoposide-cytarabine-melphalan; *TTF* thiotepa-treosulfan-fludarabine; *Bu-Cy* busulfan-cyclophosphamide; *Flu-Cy-Thi* fludarabine-cyclophosphamide-thiotepa; *TBF* thiotepa-busulfan-fludarabine; *Cy-Flu-Mel* cyclophosphamide-fludarabine-melphalan; *Bu-Flu* busulfan-fludarabine; *MW* Mann–Whitney test; *CsA/MTX* cyclosporine/methotrexate; *SRL/MPA* sirolimus/mycophenolate acid; *GCSF* granulocyte-stimulating factor; *KGF* keratinocyte growth factor; *F* Fisher exact test; *OM* oral mucositis

Descriptive analysis was performed using SPSS (IBM Corp. Released 2015, IBM SPSS Statistics for Windows, Version 23.0, Armonk, NY: IBM Corp.), and the Matrix Laboratory (MATLAB) Statistical toolbox version 2008 (MathWorks, Natick, MA, USA) was used for comparative analysis. All tests with *P* ≤ 0.05 were considered significant.

## Results

### Participants’ characteristics

Seventy-one HSCTs were performed from November 2017 to September 2019 in our hematology unit: 4 patients refused to participate in this study and 8 were ineligible. Thus, 59 patients were recruited 32 (54.2%) male and 27 (45.8%) female, with mean age 52.4 years (SD ± 12.0; range 18–71). All participants in the SG were adults and had no signs of OM at admission; 6 patients (10.2%) were smokers, 10 (16.9%) experienced OM during pre-transplant therapies, and none had a history of alcohol abuse. Most participants were married (42; 71.2%) and worked (44; 74.6%). Granulocyte-stimulating factor (GCSF) was administered to all autologous HSCT patients by day 1 post-transplant in both groups; only 1 allogeneic patient in SG was treated with GCSF from day + 18 due to infection. Main clinical information on SG and CG are showed in Table 1. No significant differences between groups are reported, including some risk factors for OM development.

### Oral mucositis

During the first step of the study, 15/19 participants (78.9%) experienced OM; one patient (5.3%) developed sOM (grade 3 WHO), and no serious AEs were recorded in the report form. This made it possible to continue patient recruitment.

At the end of the study period (22 months), 46 (78.0%) patients experienced OM: 40 (67.8%) developed mild–moderate OM (WHO grades 1–2) and 6 (10.2%) developed sOM (WHO grades 3–4). Of those who developed sOM, 4 received allograft (2 BMF and 2 HL/NHL) and 2 patients had undergone autologous HSCT (2 PD). The incidence of patients without OM was 22.0% (13 cases). The mean duration of OM (any grade) was 7.9 days (SD ± 5.8); the mean duration of sOM was 0.5 days (SD ± 1.9). OM occurred on average 4.5 days (SD ± 2.4) post-transplant and 9.1 days (SD ± 3.5) after the start of conditioning regimen. The mean time of onset of sOM was 7.8 days (SD ± 1.7) post-transplant and 11.2 days (SD ± 2.9) from the first day of conditioning.

### Historical cohort comparison

Severe OM incidence decreased more significantly in SG than in CG (10.2% vs. 28.4%, respectively; *P* < 0.01), while overall OM incidence remained unvaried (78.0% vs 80.2%; *P* = 0.74) (Table 2). No significant differences were seen in either overall or sOM time of onset with reference to HSCT and the start of conditioning. Severe OM mean duration was longer in CG (1.5 ± 3.0 vs. 0.5 ± 1.9 days; mean rank 75.9 vs. 63.1; *P* < 0.01), while there was no difference in overall duration of OM. Fewer patients in SG than in CG (41/59 vs. 77/81 patients, respectively; *P* < 0.01) developed FN, and its mean duration was shorter (4.0 ± 4.7 vs. 6.2 ± 4.5 days, respectively; *P* < 0.01) (Tables 2 and 3). No differences between the groups were found regarding neutropenia duration, length of hospital stay, maximum pain score (MPS), or the duration (days) of treatment with opioids, PN, antibiotics, or antifungal medications. The mean duration of antiviral therapy was significantly shorter in SG than in the CG (2.7 ± 7.3 vs. 9.5 ± 13.0; *P* < 0.01).

**Table 2:** Oral mucositis and febrile neutropenia

Variable			Control group *n* = 81	Study group *n* = 59	*P* value (test)
			*n* (%)	*n* (%)	
OM	No		16 (19.7)	13 (22.0%)	0.74 (C)
	Yes		65 (80.2)	46 (78.0%)	
		Mild (1–2 WHO)	42 (51.9)	40 (67.8%)	< 0.01 (C)*
		Severe (3–4 WHO)	23 (28.4)	6 (10.2%)	
Neutropenia (ANC < 500)			81 (100.0)	59 (100.0)	1.0 (C)
FN	Yes		77 (95.1)	41 (69.5)	< 0.01 (C)*
	No		4 (4.9)	18 (30.5)	

*n* number; *OM* oral mucositis; *WHO* World Health Organization; *ANC* absolute neutrophil count; *FN* febrile neutropenia

^*^Significant test

**Table 3:** Secondary outcomes comparisons

Variables		Control group			Study group			*P* value (test)
		*n*	Mean ± SD	Median (IQR)	*n*	Mean ± SD	Median (IQR)	
Onset time of OM (days)	HSCT	65	4.2 ± 3.4	4 (2–6)	45	4.4 ± 2.4	5 (3–6)	0.53 (T, W)
	SoC		10.0 ± 3.8	10 (7.75–12)		9.1 ± 3.4	9 (7–11.25)	0.19 (T)
Onset time of sOM (days)	HSCT	23	7.5 ± 3.2	7 (5–8.75)	6	7.8 ± 1.7	8 (7–9)	0.34 (MW)
	SoC		13.4 ± 4.5	13 (9.25–16)		11.2 ± 2.9	10 (10–11)	0.25 (T)
Days of OM		81	8.0 ± 6.5	8 (2–11.25)	59	7.9 ± 5.8	8 (2.25–12.75)	0.87 (MW)
Days of sOM			1.5 ± 3.0	0 (0–1.25); 75.9**		0.5 ± 1.9	0 (0–0); 63.1**	< 0.01 (MW) *
MPS			3.7 ± 2.7	4 (1–6)		3.7 ± 2.7	4 (2–5)	0.88 (MW)
Days of neutropenia			10.7 ± 6.9	9 (6–11)		11.9 ± 9.2	8 (6–12.75)	0.79 (MW)
Days of FN			6.2 ± 4.5	6 (3–8)		4.0 ± 4.7	3 (0–6)	< 0.01 (MW) *
Days of antibiotic use			11.1 ± 12.3	8 (4.75–14.25)		11.1 ± 12.0	10 (1.25–14.75)	0.97 (MW)
Days of antifungal use			9.1 ± 12.2	6 (0–12)		10.5 ± 11.6	10 (0–13.75)	0.19 (MW)
Days of antiviral use			9.5 ± 13.0	4 (0–17)		2.7 ± 7.3	0 (0–0)	< 0.01 (MW) *
Days of PN use			4.2 ± 6.6	0 (0–8)		5.0 ± 7.7	0 (0–7)	0.49 (MW)
Days of opioid use			4.8 ± 4.7	4 (0–8)		3.8 ± 6.9	0 (0–8)	0.05 (MW)
Length of stay (days)			25.7 ± 13.7	21 (17–31)		26.9 ± 13.2	22 (18–30.75)	0.35 (MW)

*SD* standard deviation; *IQR* interquartile range; *CG* control group; *SG* study group; *OM* oral mucositis; *HSCT* hematopoietic stem cell transplantation; *sOM* severe oral mucositis; *SoC* start of conditioning; *MPS* maximum pain score; *FN* febrile neutropenia; *PN* parenteral nutrition; *T t*-test; *W* Welch test for correction of unequal variances; *MW* Mann–Whitney test

^*^Significant test; **mean rank value (it was indicated if necessary)

### Adherence

**Fig. 1:**
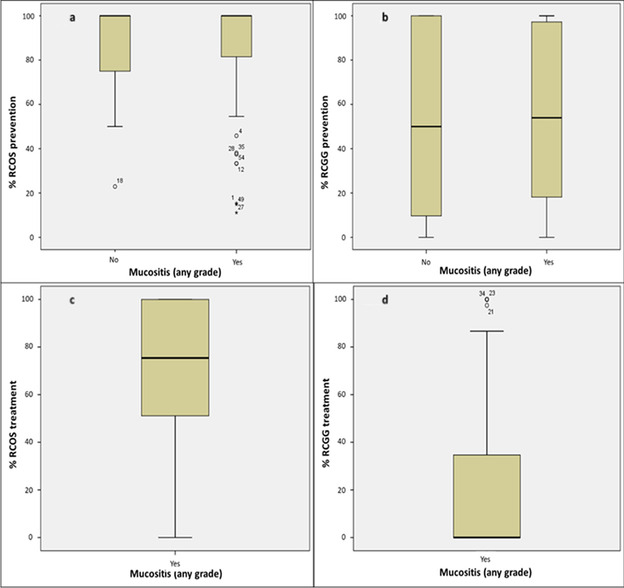
Adherence per mucositis development (any grade) during the prevention and treatment phases

**Fig. 2:**
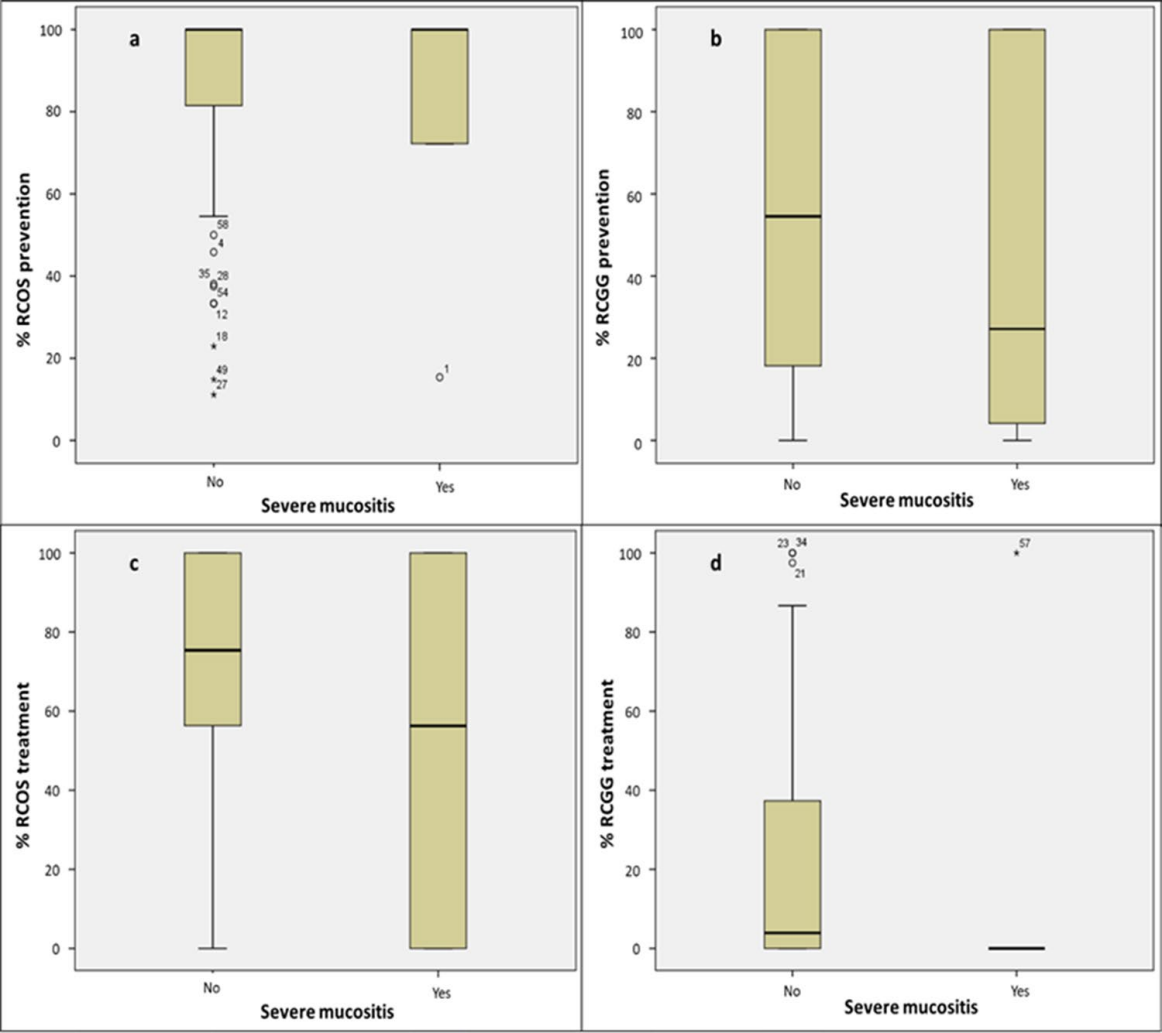
Adherence per severe oral mucositis development during the prevention and treatment phases

Overall adherence to the study protocol is described in Table 4. During the prevention phase, 17/59 (28.8%) patients were fully compliant with the oral care protocol, while 42 (71.2%) did not take at least a dose of one of the experimental products. Thirty-five (59.3%) participants were 100% compliant with the RCOS mouthwashes; the mean percentage of adherence to prevention was 84.4 (SD ± 26.9). Seventeen patients (28.8%) were 100% compliant with RCGG administration; the mean percentage of adherence was 54.3 (SD ± 39.1). Forty-six patients developed OM. In the treatment phase, adherence was lower: 7/46 (15.2%) patients were compliant with at least 3 applications per day of the study protocol, while 39 (84.8%) were not. Twenty-four (52.2%) participants were 100% compliant with RCOS mouthwashes during this phase; the mean percentage of adherence was 77.8 (SD ± 34.7); 7 (15.2%) patients were 100% compliant with RCGG administration; the mean percentage of adherence was 28.3 (SD ± 39.1). The reasons for not complete adherence were primarily clinical (64.4% of prevention days; 63.3% of treatment days) due to chemotherapy-related gastrointestinal symptoms such as nausea and vomiting, diarrhea, and taste change. Voluntary adherence discontinuation due to fatigue symptoms was adopted in 34.6% of prevention days and 36.5% of treatment days, while intolerance to the study products was in 1.0% of prevention days and not specified in 0.2% of treatment days. In general, patients compliant with the intervention during treatment had significantly higher compliance during prevention (96.9% vs. 69.5%) and for RCGG (95.9% vs. 46.8%). During the prevention phase, no significant differences in adherence to RCOS and RCGG were found between patients who subsequently developed OM and those who did not (RCOS 83.8% vs. 85.4%: RCGG 54.3% vs. 53.9%) (Fig. 1). The development of sOM was correlated with lower adherence to RCGG during prevention (43.1% vs. 55.5%) and was associated with a decrease in adherence during the treatment phase (RCOS 52.1% vs. 70.2%; RCGG 16.7% vs. 22.5%) (Fig. 2). No differences in adherence to the study protocol were found per type of transplant or underlying disease.

**Table 4:** Overall adherence to the study protocol

	Prevention (*n* = 59)				Treatment (*n* = 46)			
	100% adherence	*n*	%	Overall adherence % (± SD)	100% adherence	*n*	%	Overall adherence % (± SD)
RCOS	Y	35	59.3	84.4 (26.9)	Y	24	52.2	77.8 (34.7)
	N	24	40.7		N	22	47.8	
RCGG	Y	17	28.8	54.3 (39.1)	Y	7	15.2	28.3 (39.1)
	N	42	71.2		N	39	84.8	
Whole Protocol	Y	17	28.8	69.4 (26.4)	Y	7	15.2	53.1 (30.4)
	N	42	71.2		N	39	84.8	

*RCOS* Remargin Colostrum OS®; *RCGG* Remargin Colostrum Gastro-Gel®; *Y* Yes; *N* No

### Safety and adverse events

**Fig. 3:**
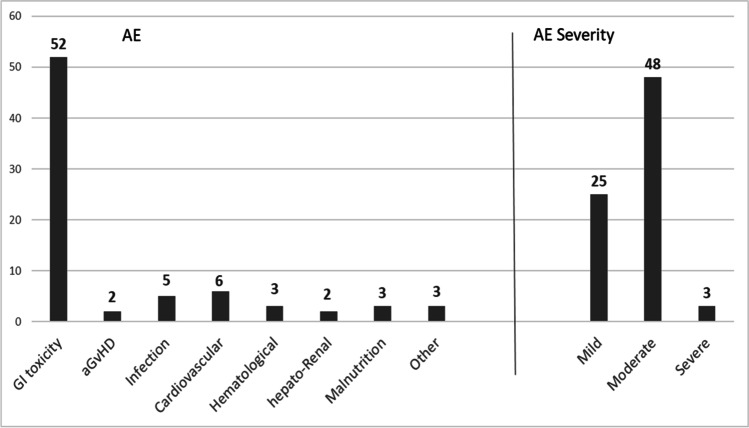
AE frequency and severity

Of the 76 AEs recorded in the case report form, 14 (18.4%) occurred and resolved before OM onset (prevention phase), 28 (36.4%) were observed after OM onset (treatment phase), and 34 (44.7%) manifested across both periods. Most AEs (52; 68.4%) were conditioning-related gastrointestinal symptoms (Fig. 3). No deviations of the monitored biochemical parameters were detected during and after the study period, and none of the registered AEs resulted correlated with the experimental oral care protocol. Weekly monitoring of galactomannan serum levels did not show alterations. At admission, 22 (37.3%) of SG patients’ oral cavities were colonized by *Candida albicans*, 36 (61.0%) were negative and 1 (1.7%) was colonized by *Streptococcus agalactiae*. On day 8 post-transplant, candida-colonized mouths decreased to 18 (30.5%), while 40 (67.8%) were negative and 1 (1.7%) was positive to *Saccharomyces cerevisiae.*

## Discussion

The safety and efficacy of BC- and AV-based oral care protocol on HSCT patients with sOM were assessed in our prospective study. Safety was monitored by analyzing data on AEs, biochemical parameters, and culture tests routinely collected during the patients’ hospital stay. The compared groups were homogeneous in terms of number of participants, sex, age, type of transplant, underlying diagnosis, stem cell source, type of cell product, conditioning regimen, immunosuppressive strategy, growth factor use, and risk factors for OM development. Despite there being no difference between groups in overall OM development, a reduction of up to 60% of sOM incidence was found in SG. In addition, sOM mean duration per group appeared shorter in SG, and a correlation between the reduction in sOM incidence and the reduction in the number of FN episodes and duration appeared evident. Rathe et al. (2020) [44] explored the effects on chemotherapy-related toxicities of daily BC dietary supplementation obtaining a reduction of OM peak of severity in the treatment group more than in the control group. However, in Rathe’s trial, as OM severity was a secondary endpoint, the risk of an underpowered sample was posed. Furthermore, the effects of BC supplementation on FN were not significant, while our study suggested a reduction effect.

Beneficial effects of bioactive milk factors on oral mucosa exposed to chemotherapy were described in a pre-clinical study on hamster [54]. Significant reduction in severity and duration of OM was reported in two studies on HSCT patients undergoing chemotherapy-based conditioning regimens where whey proteins were administered as dietary supplements (systemic effect) [55] and as mouthwashes (topical effect) [56].

BC antibacterial activity conferred by lactoferrin, lactoperoxidase, and a variety of immunoglobulins [57–59], combined with the antimicrobial properties of AV against the *Candida* species [60–62] and type 2 herpes simplex [63], could explain some of our significant findings, such as the reduction in FN (episodes and duration) and the reduced use of antiviral medication in SG. Weak evidences of the benefits of BC on the integrity of the mucosal barrier, reducing intestinal bacteria translocation, have been reported [33, 34], and AV’s in vitro antiviral action has been described [63]. The immunomodulatory and anti-inflammatory effects of AV were provided by the stimulation of macrophages and modulating cyclooxygenase activation pathway [47, 63–65], which is fundamental to OM pathobiology [11]. BC contains a range of cytokines and other non-antimicrobial substances that together modulate inflammation and maintain or improve host response under different immune system exposures [40, 66].

Dietary supplementation of BC may trigger immunological events that lead to systemic effects [33]. However, the limited patient adherence to RCGG intake gave rise to several doubts on the real potency of any systemic effect in this study.

The beneficial effects of AV and of BC on wound care have been suggested by some preclinical studies [41, 67, 68]. Their effects on mucocutaneous issues such as pain reduction, wound healing acceleration, stomatitis healing, and QoL improvement are well known [42, 43, 51, 52]. It has been suggested that some components of BC, such as nucleotides, epidermal growth factor (EGF), and insulin-like growth factor-1 (IGF-1), promote mucocutaneous cellular growth and also help repair gene impairment [32]. The tissue regenerative properties of AV are due to its component mannose-6-phosphate (M6P), which plays a fundamental role in extracellular matrix remodelling as well as in increasing proliferation of fibroblasts and collagen and in producing some fundamental substances such as hyaluronic acid [69–71].

In our study, the anti-inflammatory and antimicrobial properties and the tissue healing capability of AV and BC described above were beneficial during the ulcerative phase of OM, when the oral microbial flora plays a fundamental role in amplifying gene signals, accelerating tissue damage, and increasing inflammation, pain, and the risk of systemic infections [11]. Therefore, the effects of the oral care protocol were observed primarily on sOM development and duration. Although not statistically significant, the observed reduction in the number of days of opioids use in SG might confirm this hypothesis.

The reduced compliance to the oral care protocol due to factors such as chemotherapy-related toxicities may be a limiting factor of this study. Patients affected by nausea, vomiting, and/or taste change had difficulties taking study products due to their consistence (RCGG) and/or flavor (RCOS). In particular, difficulties in RCGG swallowing were reported by these patients, especially after mucositis onset. Although our results suggest a strong effect, the oral care protocol included different strategies, such as topical and systemic interventions, which precluded any consideration on the effect of the individual products. The comparison with the historical control group may have involved the change in some undetectable variables, leading to result biases. Furthermore, the strategies for supportive therapy and care may have varied because of healthcare professionals’ decisions or patients’ needs.

To our knowledge, this was the first study on the combination of BC and AV for the prevention and treatment of OM in oncology setting. The oral care protocol investigated in this study showed significant results on sOM incidence without any significant AEs. Our findings may be explained by the activity of the multiple bioactive substances composing BC and AV, and secondary findings seem to confirm the antimicrobial effects of both compounds, as already suggested in the literature [33, 34, 47, 60]. However, the study design and some limitations suggest caution when interpreting these results. A randomized controlled trial is necessary to provide evidence in favor or against the use of this approach in clinical practice. It is implemented at our institute.

## Data Availability

The datasets generated and/or analyzed during the current study are available from the corresponding author on reasonable request.
